# Inverted U-shaped relationship between HbA1c and diabetic retinopathy in diabetic patients: a cross-sectional study

**DOI:** 10.1186/s12886-025-04079-8

**Published:** 2025-05-13

**Authors:** Juan Ling, ZhuoLin Xie, XiaoJie Chen, Di Ling, XingLin Chen, XiangXia Luo

**Affiliations:** 1 Clinical College of Chinese Medicine, Gansu Province, Gansu University of Chinese Medicine, Lanzhou, 730030 China; 2 Present Address: Gansu Provincial Hospital, Lanzhou, 730030 China; 3 Present Address: Gansu Province, Gansu Provincial Hospital of TCM, Lanzhou, 730050 China; 4 The Third People’s Hospital of Gansu Province, Gansu Province, Lanzhou, 730050 China; 5 Department of Geriatrics, Union Hospital, Tongji Medical College, Huazhong University of Science and Technology, Wuhan, China; 6 Department of Epidemiology and Biostatistics, X&Y Solutions Inc, Empower U, Boston, USA

**Keywords:** HbA1c, Diabetic retinopathy, Inverted U-Shaped Relationship, Cross-Sectional

## Abstract

**Background:**

Diabetic retinopathy (DR) is a leading cause of blindness among adults with diabetes. Glycated hemoglobin A1C (HbA1C) is a critical biomarker for long-term glycemic control and has been closely associated with the risk of developing DR. However, the relationship between HbA1C and DR remains complex and multifaceted, with limited research exploring the nonlinear aspects of this association. This study aims to investigate the nonlinear relationship between HbA1C and DR, providing insights into their association and informing clinical interventions.

**Objective:**

Many studies have indicated that HbA1C is positively correlated with DR. However, although elevated HbA1C is common in patients with DR, its relationship with DR remains controversial. Our study aimed to investigate the nonlinear relationship between HbA1c and DR, thereby accurately elucidating their association and providing a basis for clinical interventions.

**Methods:**

This study is the second analysis based on a cross-sectional studv. A total of 2,001 patients with type 2 Diabetes Mellitus (T2DM) visited the diabetic clinic in the Internal Medicine outpatient departments of two hospitals in southern Taiwan between April 2002 and November 2004 were included in this analysis. Demographic and clinical data were collected, and HbA1c levels were measured. The association between HbA1c and DR was analyzed using multivariate logistic regression, adjusting for potential confounders, and the potential nonlinear correlation was explored with a smooth curve fitting approach.

**Results:**

The fully-adjusted model showed that HbA1c positively correlated with DR (OR:1.13, 95%CI: 1.05–1.22). However, an inverted U-shaped association between them was observed by applying the smooth curve fitted method. The inflection point of HbA1c (9.4%) was calculated by utilizing the two-piecewise logistic regression model. In the subgroup analysis, the inverted U-shaped nonlinear correlation between HbA1c and DR was also found in age, sex and BMI.

**Conclusions:**

HbA1C and DR have an inverted U-shaped relationship, with a peak at an HbA1C of 9.4% in the early phase of DR. After this peak, HbA1C decreases as DR increases. These results have crucial implications for DR patients. The findings also offer insights for public health policy, highlighting the necessity of regular screening and intervention for diabetic patients. Future research should further explore the mechanisms linking HbA1c to DR and consider individualized management strategies for different populations to effectively mitigate the burden of DR.

**Supplementary Information:**

The online version contains supplementary material available at 10.1186/s12886-025-04079-8.

## Introduction

Diabetic retinopathy (DR) is one of the most common complications among patients with diabetes mellitus and a leading cause of blindness in adults. According to the International Diabetes Federation (IDF), approximately 463 million people worldwide have diabetes, with the prevalence of DR reaching as high as 30% to 40% among diabetic patients [1]. This staggering statistic underscores the urgent need for effective screening, prevention, and treatment strategies, particularly as the global diabetes epidemic continues to escalate. The World Health Organization (WHO) projects that by 2045, the number of individuals with diabetes will rise to 700 million, further exacerbating the burden of DR and its associated healthcare costs [2]. The pathophysiology of DR is complex and multifactorial, involving a cascade of biochemical and cellular changes triggered by chronic hyperglycemia. Prolonged elevated blood glucose levels lead to the accumulation of advanced glycation end-products (AGEs), which contribute to oxidative stress and inflammation within the retinal microenvironment. These processes result in the dysfunction of retinal endothelial cells, increased vascular permeability, and the formation of microaneurysms, ultimately leading to retinal ischemia and neovascularization [3, 4].

Glycated hemoglobin A1 C (HbA1 C) is produced through the non-enzymatic glycation of hemoglobin. For individuals with diabetes mellitus, HbA1 C serves as a therapeutic target for adjusting glucose-lowering treatments, as it shows a significant correlation with the risk of developing microvascular complications related to diabetes mellitus [5]. Furthermore, HbA1 C displays lower intra-individual variability when compared to both fasting glucose and 2-h post-challenge glucose levels after an oral glucose tolerance test, and it can be assessed without requiring fasting [6]. For these reasons, HbA1 C has been endorsed as a diagnostic criterion for diabetes [7].As an important indicator of long-term blood glucose control, HbA1 C has been closely associated with the risk of developing DR. Numerous studies have demonstrated that elevated HbA1 C levels are positively correlated with an increased incidence of DR [8–10]. This may be attributed to the microvascular damage and oxidative stress caused by chronic hyperglycemia. Additionally, HbA1 C levels may also participate in the development of DR through mechanisms involving the impact on retinal microcirculation, neurotrophic factors, and immune function [3, 11]. Therefore, HbA1 C is not only a crucial indicator for the diagnosis and treatment of diabetes, but also a key biomarker for predicting and evaluating the risk of DR. Further exploring the complex relationship between HbA1 C and DR can contribute to a deeper understanding of the pathogenesis of diabetic complications, providing a basis for clinical prevention and management.

However, the association between DR and HbA1c is a subject of ongoing debate, characterized by its complexity and multifactorial nature. Seminal cohort studies, such as the UK Prospective Diabetes Study (UKPDS) and the Diabetes Control and Complications Trial (DCCT), have robustly established that a 1% reduction in HbA1c is associated with a 30%− 40% decrease in DR risk, underscoring the critical role of glycemic control in mitigating DR progression [12, 13]. However, a significant proportion of patients (approximately 29.6%) with well-controlled HbA1c levels (< 7%) still develop incident DR, suggesting that additional metabolic factors, such as ethanolamine deficiency, may play a pivotal role in DR pathogenesis [14]. The impact of HbA1c variability (VVV) on DR remains a contentious issue. Emerging evidence indicates that VVV may act as an independent risk factor, with a 48% increase in DR risk observed per 1% rise in HbA1c standard deviation (SD) [15], and its influence on DR progression may even surpass that of the average HbA1c level [16]. In contrast, a large-scale Japanese cohort study involving 5,898 patients with type 2 diabetes mellitus (T2DM) found no significant association between the HbA1c coefficient of variation (CV) and DR [17], highlighting the potential limitations of generalizability due to population heterogeneity and methodological discrepancies, such as differences in diabetes subtypes or lack of assay standardization. Furthermore, the effects of rapid HbA1c reduction on DR progression remain controversial. The 2024 EURETINA study reported that a decline in HbA1c of ≥ 1.5% within 3 months may exacerbate DR progression by impairing retinal hemodynamic adaptation [18]. Conversely, a retrospective analysis of 1,150 patients demonstrated no association between rapid HbA1c reduction (> 1.5% within 12 months) and the progression of mild to moderate non-proliferative diabetic retinopathy (NPDR) [19], suggesting that disease stage or glycemic velocity thresholds may modulate this relationship. Collectively, these findings underscore the complexity of HbA1c-DR interactions and controversial, emphasizing the need for further research to elucidate the underlying mechanisms and optimize therapeutic strategies.

Therefore, this study aimed to investigate the nonlinear association between HbA1c and DR through a cross-sectional study of diabetic patients. We analyzed the relationship between different HbA1c levels and the occurrence of DR while considering various potential confounding factors. Through this research, we hope to provide more scientific evidence for the clinical management of diabetic patients and inform public health policies to reduce the incidence of DR.

## Methods

### Study population

In this secondary analysis, we utilized data derived from the study conducted by Chen SC et al. [20], published in the esteemed journal PloS One (10.1371/journal.pone.0134718). This dataset was made freely available for download, adhering to principles of open-access research. The investigation carried out by Chen SC et al. represented a comprehensive survey conducted across diabetes clinics within the Internal Medicine outpatient departments of two hospitals located in southern Taiwan, covering the timeframe from April 2002 to November 2004.

The study initially recruited a total of 2001 participants, which included 858 males and 1143 females, providing a solid demographic foundation for analysis. The average age of the participants was 64.1 years, accompanied by a standard deviation of ± 11.3 years, indicating a diverse age range that is pertinent to the prevalence and management of diabetes in this population.

### Ethics Statement

The original study has already obtained the necessary Ethics Statement and the study was conducted in accordance with the Declaration of Helsinki, adhering to both international ethical standards and local regulations of previously study [20]. The Institutional Review Board of Kaohsiung Medical University Hospital approved the study protocol (approval number: KMUHIRB-E- 20150029). Before participating, all subjects provided written informed consent, which included permission for the publication of their anonymized clinical data. Further information can be found at 10.1371/journal.pone.0134718.

### Variables

Demographic and medical information, including age, gender, and co-morbidities, were collected from patients’ medical records and interviews. Body mass index (BMI) was determined by dividing weight in kilograms by the square of height in meters. Laboratory tests on fasting blood samples were conducted using an autoanalyzer (Roche Diagnostics GmbH, D- 68298 Mannheim COBAS Integra 400). Serum creatinine levels were measured with the compensated Jaffé (kinetic alkaline picrate) method on a Roche/Integra 400 Analyzer (Roche Diagnostics, Mannheim, Germany), using a calibrator traceable to isotope-dilution mass spectrometry [21]. The estimated glomerular filtration rate (eGFR) was calculated based on the 4-variable equation from the Modification of Diet in Renal Disease (MDRD) study [22]. Urine albumin and creatinine concentrations were assessed from a spot urine sample utilizing the COBAS Integra 400 plus autoanalyzer (Roche Diagnostics, North America), with microalbuminuria defined as a urine albumin-to-creatinine ratio of ≥ 30 mg/g. Blood samples were obtained within one month prior to the measurement of the ankle-brachial index (ABI).

### DR confirmation

DR confirmation was performed by certified ophthalmologists based on comprehensive eye examinations. Patients underwent funduscopy and optical coherence tomography (OCT) to detect characteristic retinal lesions such as microaneurysms, hemorrhages, and exudates. The diagnosis was established according to the International Clinical Diabetic Retinopathy Disease Severity Scale, confirming the presence or absence of DR [23].

### Statistical analysis

Categorical variables were expressed as counts and percentages, whereas continuous variables were reported as either means with standard deviations (SD) or medians with interquartile ranges (25 th to 75 th percentiles), based on the data distribution. *P*-values for continuous variables were obtained through weighted linear regression models, while the chi-square test was applied to categorical data. The association between DR and HbA1c levels was analyzed using multivariate logistic regression and smooth curve fitting, accounting for relevant clinical covariates. An inflection point was detected using a recursive algorithm. For instances of non-linearity, a weighted two-piecewise logistic regression model was utilized. Statistical analyses were performed using EmpowerStats software (http://www.empowerstats.com) and R version 4.1.1, with a *p*-value of less than 0.05 considered statistically significant.

## Results

In this study, a total of 2001 patients with type 2 diabetes were enrolled, consisting of 1300 individuals (65.0%) without diabetic retinopathy (non-DR) and 701 individuals (35.0%) with DR. The mean age of the overall study population was 64.0 ± 11.3 years. Additionally, the mean body mass index (BMI) was recorded, along with a mean systolic blood pressure (SBP) of 135.4 ± 18.9 mmHg, a mean abdominal circumference (AC) of 78.1 ± 10.81 cm, and a mean cholesterol level of 185.3 ± 38.8 mg/dL. Further details regarding the baseline characteristics of patients with and without DR are presented in Table 1.

**Table 1:** Baseline Characteristics of the study participants (*n* = 2001)

Characteristic	No Retinopathy (*N* = 1300)	Retinopathy (*N* = 701)	Standardized Difference (95% CI)	*P*-value	*P*-value*
Age(years)	63.3 ± 11.8	65.4 ± 10.2	0.2 (0.1, 0.3)	< 0.001	< 0.001
BMI(kg/m^2^)	25.8 ± 3.6	25.9 ± 3.4	0.0 (− 0.1, 0.1)	0.505	0.391
SBP(mmHg)	133.7 ± 18.4	137.1 ± 19.3	0.2 (0.1, 0.3)	< 0.001	< 0.001
DBP(mmHg)	77.8 ± 11.1	77.7 ± 11.7	0.0 (− 0.1, 0.1)	0.831	0.606
AC(cm)	77.1 ± 11.9	79.1 ± 9.6	0.2 (− 0.2, 0.5)	0.309	0.532
Laboratory parameters					
Cholesterol(mg/dL)	186.5 ± 38.3	184.1 ± 39.3	0.1 (− 0.0, 0.2)	0.184	0.06
Triglycerides(mg/dL)	154.9 ± 136.2	155.0 ± 134.1	0.0 (− 0.1, 0.1)	0.995	0.394
LDL(mg/dL)	104.8 ± 28.5	103.4 ± 28.4	0.1 (− 0.0, 0.1)	0.284	0.218
HDL(mg/dL)	50.1 ± 13.2	48.5 ± 12.7	0.1 (0.0, 0.2)	0.007	0.008
Creatine(mg/dL)	1.0 ± 0.4	1.1 ± 0.4	0.2 (0.1, 0.3)	< 0.001	< 0.001
eGFR(mL/min/1.73 m^2^)	70.4 ± 19.4	65.4 ± 19.8	0.3 (0.2, 0.3)	< 0.001	< 0.001
ABI	1.1 ± 0.1	1.1 ± 0.1	0.0 (− 0.0, 0.1)	0.346	0.915
Sex			0.0 (− 0.1, 0.1)	0.565	-
Male	748 (57.5%)	394 (56.2%)			
Female	552 (42.5%)	307 (43.8%)			
ACR30			0.3 (0.2, 0.4)	< 0.001	-
No	911 (70.1%)	392 (55.9%)			
Yes	389 (29.9%)	309 (44.1%)			
Stroke			0.1 (0.0, 0.2)	0.004	-
No	1249 (96.1%)	653 (93.2%)			
Yes	51 (3.9%)	48 (6.8%)			
Ischaemic Heart Disease			0.1 (0.0, 0.2)	0.009	-
No	1102 (84.8%)	562 (80.2%)			
Yes	198 (15.2%)	139 (19.8%)			
Medications					
ACEI and/or ARB use (%)	913 (70.6%)	554 (79.1%)	0.2 (0.1, 0.3)	< 0.001	
β-blocker use (%)	288 (22.3%)	178 (25.4%)	0.1 (− 0.0, 0.2)	< 0.001	
Calcium channel blocker use (%)	446 (34.5%)	335 (47.9%)	0.3 (0.2, 0.4)	< 0.001	
Diuretic use (%)	547 (42.3%)	370 (52.9%)	0.2 (0.1, 0.3)	< 0.001	

Mean ± SD or Median (25 th, 75 th percentile) for continuous variables; *P* value was calculated by weighted linear regression model. % for categorical variables; *P* value was calculated by weighted chi-square test; ABI:ankle-brachial index; HDL:high-density lipoprotein; LDL: low-density lipoprotein; eGFR: estimated glomerular filtration rate; ACR: albumin-to-creatinine ratio; BMI:Body Mass Index; SBP:Systolic Blood Pressure; AC: Abdominal Circumference; DBP: Diastolic Blood Pressure

Table 2 demonstrates a significant association between HbA1c levels and the prevalence of DR. In Model 1, without any covariate adjustments, each 1% increase in HbA1c was linked to a 7% increase in the odds of DR (OR 1.07, 95% CI: 1.01–1.13, *P* = 0.0230). This association strengthened in Model 2, which adjusted for sex and age, resulting in an odds ratio of 1.09 (95% CI: 1.03–1.15, *P* = 0.0022). In Model 3, which accounted for multiple covariates including age, sex, BMI, SBP, DBP, AC, cholesterol, triglycerides, LDL, HDL, ischemic heart disease, ACEI and/or ARB use, β-blocker use, Diuretic use, Calcium channel blocker use and ABI, the odds ratio further increased to 1.13 (95% CI: 1.05–1.22, *P* = 0.0012). When HbA1c was analyzed in tertiles, the low tertile served as the reference group, with the middle tertile showing an increased odds of DR (OR 1.34, 95% CI: 1.06–1.69, *P* = 0.0140) in Model 1, which remained significant in Model 2 (OR 1.33, 95% CI: 1.06–1.70, *P* = 0.0136) and Model 3 (OR 1.36, 95% CI: 1.07–1.74, *P* = 0.0119). The high tertile exhibited the strongest association, with an OR of 1.56 (95% CI: 1.24–1.96, *P* < 0.0001) in Model 1, increasing to 1.68 (95% CI: 1.34–2.12, *P* < 0.0001) in Model 2, and reaching 1.80 (95% CI: 1.38–2.35, *P* < 0.0001) in Model 3. These findings highlight a significant and progressive relationship between HbA1c levels and the risk of developing DR.

**Table 2:** Association between HbA1 C and DR

HbA1 C (%)	Model 1 [OR (95% CI), P]	Model 2 [OR (95% CI), P]	Model 3 [OR (95% CI), P]
HbA1 C	1.07 (1.01, 1.13), 0.0230	1.09 (1.03, 1.15), 0.0022	1.13 (1.05, 1.22), 0.0012
HbA1 C(tertile)			
Low	1	1	1
Middle	1.34 (1.06, 1.69), 0.0140	1.33 (1.06, 1.70), 0.0136	1.36(1.07, 1.74), 0.0119
High	1.56 (1.24, 1.96), 0.0001	1.68 (1.34, 2.12), < 0.0001	1.80 (1.38, 2.35), < 0.0001

Model 1: no covariates were adjusted; Model 2: sex and age were adjusted; Model 3: age, sex, BMI, SBP, DBP, AC, Cholesterol, Triglycerides, LDL, HDL, Ischaemic Heart Disease, ACEI and/or ARB use, β-blocker use, Diuretic use, Calcium channel blocker use and ABI were adjusted

Table 3 presents the association between HbA1c levels and DR, stratified by sex, age, ACR30, ischemic heart disease, and stroke. In Model 1, males showed a significant association with an odds ratio (OR) of 1.08 (95% CI: 1.01–1.17, *P* = 0.0329), while females did not demonstrate a significant association (OR 1.04, 95% CI: 0.96–1.13, *P* = 0.2947). Age stratification revealed that individuals aged 69.9–96.4 years had a significant association (OR 1.16, 95% CI: 1.04–1.29, P = 0.0093), while younger age groups did not show significant results. Regarding ACR30, those with a positive ACR30 had an OR of 1.10 (95% CI: 1.01–1.19, *P* = 0.0348) in Model 1, increasing to 1.24 (95% CI: 1.10–1.40, *P* = 0.0004) in Model 3. In terms of ischemic heart disease, individuals without the condition had an OR of 1.05 (95% CI: 0.99–1.11, *P* = 0.1331), while those with ischemic heart disease had a significant association (OR 1.25, 95% CI: 1.06–1.46, *P* = 0.0065) in Model 1. Stroke stratification showed no significant association in those with a history of stroke (OR 1.10, 95% CI: 0.87–1.41, *P* = 0.4279), while those without stroke had an OR of 1.06 (95% CI: 1.00–1.12, *P* = 0.0350). These findings indicate that HbA1c levels are significantly associated with the risk of developing DR, particularly in specific subgroups.

**Table 3:** Association between HbA1 C and DR, stratified by Sex, age, ACR30, Ischaemic Heart Disease and stroke

	Model 1 [OR (95% CI), P]	Model 2 [OR (95% CI), P]	Model 3 [OR (95% CI), P]
Stratified by Sex			
Male	1.08 (1.01, 1.17),0.0329	1.11 (1.03, 1.20), 0.0074	1.16 (1.04, 1.28), 0.0049
Female	1.04 (0.96, 1.13),0.2947	1.07 (0.99, 1.16), 0.1082	1.11 (0.99, 1.24), 0.0640
Stratified by Age			
19.8–59.5 years	1.05 (0.96, 1.14), 0.3165	1.05 (0.96, 1.14), 0.3147	1.09 (0.96, 1.24), 0.1816
59.6–69.8 years	1.07 (0.97, 1.18), 0.2046	1.07 (0.97, 1.18), 0.1961	1.07 (0.94, 1.22), 0.3040
69.9–96.4 years	1.16 (1.04, 1.29), 0.0093	1.16 (1.04, 1.29), 0.0088	1.29 (1.12, 1.49), 0.0004
Stratified by ACR30			
No	1.00 (0.93, 1.08), 0.9996	1.00 (0.93, 1.08), 0.9848	1.03 (0.93, 1.14), 0.5544
Yes	1.10 (1.01, 1.19), 0.0348	1.09 (1.00, 1.19), 0.0438	1.24 (1.10, 1.40), 0.0004
Stratified by Ischaemic Heart Disease			
No	1.05 (0.99, 1.11), 0.1331	1.05 (0.99, 1.11), 0.1322	1.11 (1.03, 1.20), 0.0102
Yes	1.25 (1.06, 1.46), 0.0065	1.25 (1.07, 1.47), 0.0061	1.27 (1.02, 1.58), 0.0015
Stratified by Stroke			
Yes	1.10 (0.87, 1.41), 0.4279	1.10 (0.86, 1.40), 0.4677	1.01 (0.71, 1.45), 0.9411
No	1.06 (1.00, 1.12), 0.0350	1.06 (1.00, 1.12), 0.0352	1.14 (1.05, 1.23), 0.0009

Subgroup analyses stratified by sex, age, ACR30, Ischaemic heart disease and stroke, adjusted for BMI, SBP, DBP, AC, Cholesterol, Triglycerides, LDL, HDL, ACEI and/or ARB use, β-blocker use, Diuretic use, Calcium channel blocker use and ABI were adjusted

**Fig. 1:**
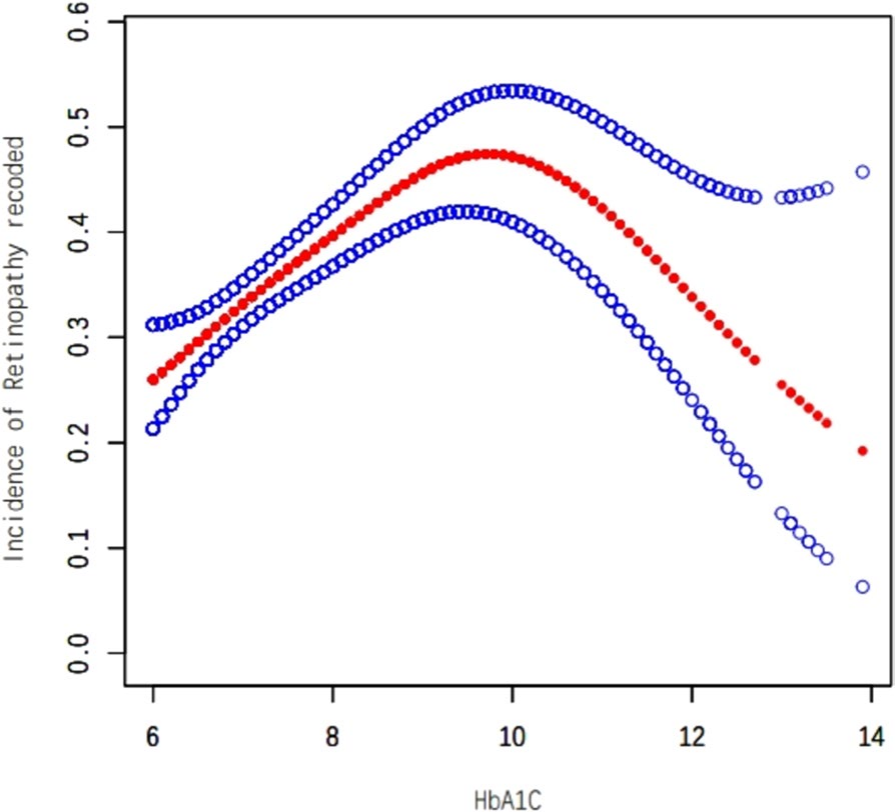
The association between HbA1 C and DR. Red line represents the smooth curve. Blue bands represent the 95% of confidence interval. Age. Sex, BMI, SBP, DBP, AC, Cholesterol, Triglycerides, LDL, HDL, Ischaemic Heart Disease, ACEI and/or ARB use,**β**-blocker use, Diuretic use, Calcium channel blocker use and ABI were adjusted

**Fig. 2:**
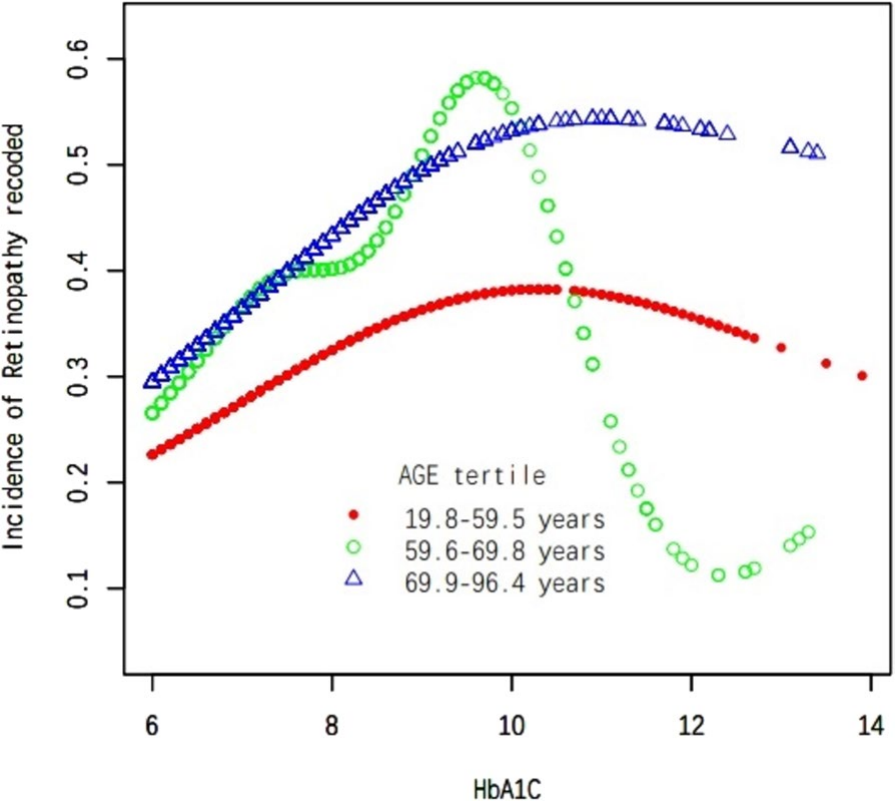
Subgroup analysis stratified by age. Sex, BMI, SBP, DBP, AC, Cholesterol, Triglycerides, LDL, HDL, Ischaemic Heart Disease, ACEI and/or ARB use,**β**-blocker use, Diuretic use, Calcium channel blocker use and ABI were adjusted

**Fig. 3:**
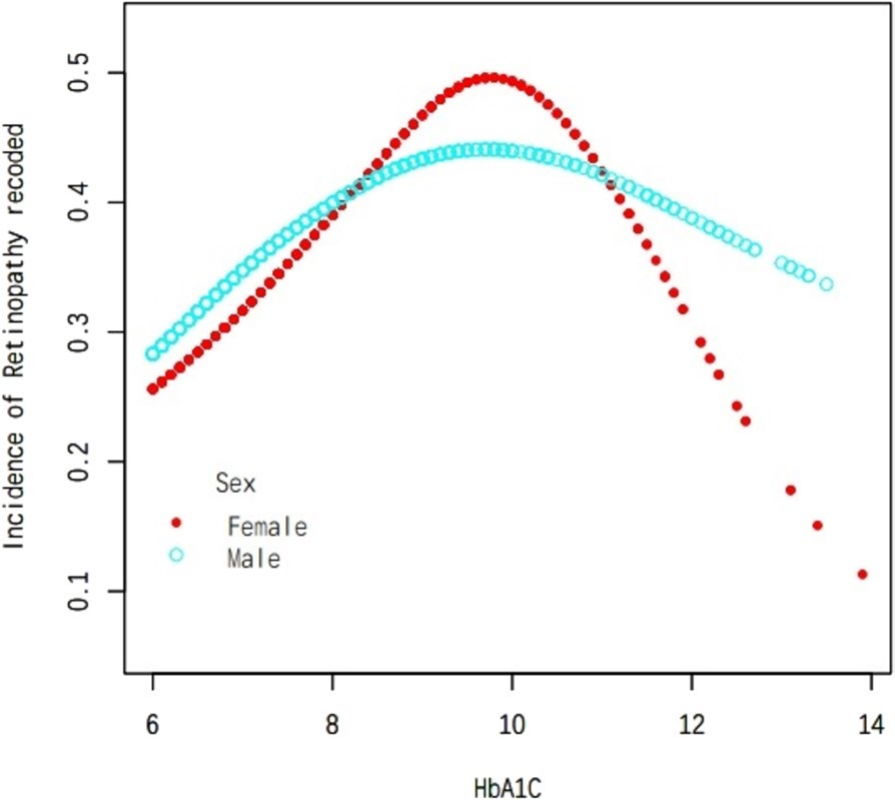
Subgroup analysis stratified by sex. Age, BMI, SBP, DBP, AC, Cholesterol, Triglycerides, LDL, HDL, Ischaemic Heart Disease, ACEI and/or ARB use,**β**-blocker use, Diuretic use, Calcium channel blocker use and ABI were adjusted

**Fig. 4:**
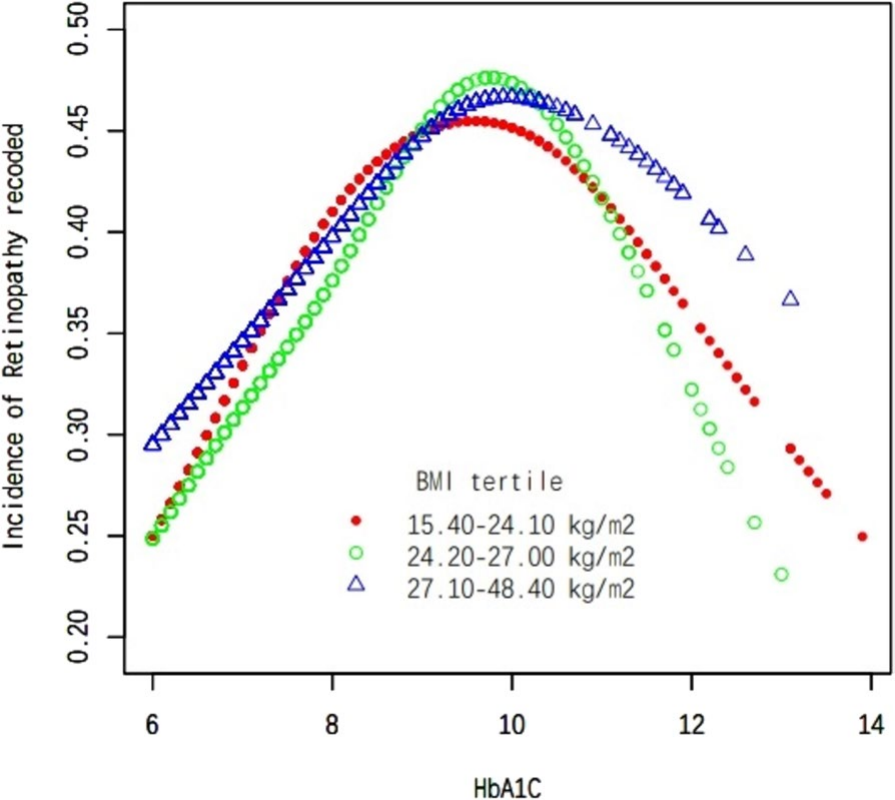
Subgroup analysis stratified by BMI. Age, sex, SBP, DBP, AC, Cholesterol, Triglycerides, LDL, HDL, Ischaemic Heart Disease, ACEI and/or ARB use, β-blocker use, Diuretic use, Calcium channel blocker use and ABI were adjusted

In this study, we employed weighted generalized additive models and smooth curve fitting to address the nonlinear correlation between HbA1 C and DR and to validate the outcomes. Smooth curve fitting is an important method for studying the nonlinear relationships between risk factors and diseases, and it has been widely adopted in numerous studies to investigate the nonlinear associations between risk factors and the risk of various diseases. The inflection points in a smooth curve are particularly valuable for public health policymakers in developing disease prevention strategies. We discovered an inverted U-shaped correlation between HbA1 C and DR (Fig. 1). In addition, in the subgroup analysis, we alse found an inverted U-shaped nonlinear relationship between HbA1 C and DR in age, sex and BMI (Figs. 2, 3 and 4). The results of the inflection points are indicated in Table 4.

**Table 4:** Threshold effect analysis for the relationship between HbA1 C and DR

Models	Incidence of retinopathy Adjusted OR (95%CI)	*P*-value
Model I		
One line slope	1.11 (1.03, 1.20)	0.0045^*^
Model II		
Turning point (K)	9.4	
< 9.4 slope 1	1.28 (1.16, 1.42)	< 0.0001^*^
> 9.4 slope 2	0.81 (0.68, 0.98)	0.0270^*^
Slope 2 – Slope 1	0.64 (0.51, 0.80)	0.0001^*^
Predicted at 9.4	− 0.11(− 0.32, 0.10)	
LRT test	< 0.001^#^	

Data were presented as OR (95%CI) *P*-value; Model I, linear analysis; Model II, non-linear analysis. LRT test, Logarithmic likelihood ratio test.(*p*-value < 0.05 means Model II is significantly different from Model I, which indicates a non-linear relationship; adjust for age, sex, SBP, DBP, AC, Cholesterol, Triglycerides, LDL, HDL, Ischaemic Heart Disease, ACEI and/or ARB use, β-blocker use, Diuretic use and ABI. *, *p* < 0.05. #, indicates that Model II is significant different from Model I

## Discussion

In this study, we investigated the nonlinear association between HbA1c levels and the prevalence of DR among a large sample of 2001 patients diagnosed with T2DM across two hospitals in southern Taiwan. The study’s design is commendable due to its substantial sample size and the cross-sectional approach, which allows for a comprehensive analysis of the relationship between glycemic control and DR.

To the best of our knowledge, this is the first study to explore the nonlinear relationship between HbA1c and DR. Our core findings reveal that each 1% increase in HbA1c is associated with a 7% increase in the odds of developing DR (odds ratio [OR] 1.07; 95% confidence interval [CI]: 1.01–1.13, *P* = 0.0230). This association strengthens when adjusted for age and sex, resulting in an OR of 1.09 (95% CI: 1.03–1.15, *P* = 0.0022) and 1.13 (95% CI: 1.05–1.22, *P* = 0.0012). Our findings are not entirely consistent with previous studies that have demonstrated a significant association between elevated HbA1c levels and the risk of developing DR. Specifically, the UK Prospective Diabetes Study (UKPDS 35) observed that for each 1% increase in HbA1c, there was a 21% increased risk of microvascular complications, including DR [12]. The UKPDS was a large-scale prospective observational study involving 3,642 patients with T2DM, emphasizing the importance of glycemic control in preventing DR. In comparison to the UKPDS, our cross-sectional study reveals a significant inverted U-shaped relationship between HbA1c levels and the risk of DR. The risk of DR peaks at an HbA1c level of 9.4%, after which it begins to decline with further increases in HbA1c levels.The discrepancies between this study and the findings of UKPDS 35 can be attributed to several factors. Firstly, differences in sample characteristics, such as sample source, age distribution, and duration of diabetes, can influence study outcomes. Secondly, variations in research design and methodology, including the distinction between prospective and retrospective designs, observation periods, and data collection and analysis techniques, may also impact the consistency of results. Additionally, the level of control for confounding factors, as well as the choice of statistical analysis methods—including statistical models, variable selection, and data processing techniques—can further contribute to the differences observed between this study and UKPDS 35. Understanding these factors is crucial for guiding future research in this area.

The results of this study showed a significant inverted U—shaped link between HbA1c levels and DR risk, peaking at 9.4% HbA1c. This finding is key for clinical and public health policy. When HbA1c is around 9.4%, DR risk is highest and then declines with further HbA1c increases. This might be due to metabolic memory [24], where long—term hyperglycemia forms a metabolic memory that sustains DR risk even after glycemic control. At very high HbA1c levels, this memory is already established, so higher HbA1c adds little to the risk. Also, hyperglycemia-induced microvascular damage [25] is partly irreversible. Once HbA1c is high, the damage is severe, and extra increases in HbA1c cause limited additional harm. Moreover, chronic hyperglycemia causes ongoing oxidative stress and inflammation [26]. When HbA1c exceeds 9.4%, these processes might plateau, so further HbA1c increases don’t worsen them much. This finding is also crucial for clinical and public health policy, aiding targeted intervention strategies. The early screening and monitoring of HbA1c levels can effectively identify high-risk patients, facilitating early intervention and reducing the incidence of DR. Clinically, healthcare providers can optimize diabetes management using these results. Patients near the 9.4% HbA1c threshold need closer monitoring and timely intervention. Regular HbA1c testing helps spot DR high-risk cases early, enabling prompt ophthalmologist referral for comprehensive eye exams, early DR diagnosis and treatment, and vision protection. Our results back personalized treatment based on individual HbA1c levels. For patients above 9.4%, consider aggressive glycemic control with complication monitoring. For those below, balance glycemic control and hypoglycemia risk. This personalized approach improves outcomes and reduces side effects. For public health policy, the 9.4% HbA1c threshold can inform clinical guideline updates, DR screening, and diabetes management recommendations, standardizing care and ensuring evidence—based interventions. Authorities can develop more effective screening programs by integrating this threshold, optimizing resource allocation, and focusing on those most likely to benefit, thus enhancing public health initiative efficiency and cost—effectiveness. Moreover, our study highlights the importance of preventive diabetes care. Public health campaigns should stress maintaining optimal HbA1c to prevent DR. Educational programs for healthcare professionals and the public can boost awareness of glycemic control’s impact on eye health, promoting better diabetes self—management.

This study demonstrates significant strengths that enhance its scientific value and clinical applicability. Firstly, the research design utilized a large sample size, enrolling 2,001 T2DM, which provides a solid foundation for the statistical significance of the results. Secondly, advanced data analysis strategies, including multivariate logistic regression and smooth curve fitting, were employed to explore the relationship between HbA1c and DR in depth. This approach not only accounted for potential confounding factors but also identified nonlinear relationships, offering a more precise risk assessment. Additionally, the comprehensive recording of baseline characteristics, including age, sex, BMI, blood pressure, and cholesterol levels, enhances the interpretability and reliability of the findings. Finally, the use of a recursive algorithm to identify inflection points and a weighted two-piecewise logistic regression model in cases of non-linearity adds flexibility and accuracy to the analysis. In summary, the design and analytical strategies of this study provide important insights into the complex relationship between HbA1c and DR, carrying significant clinical implications.

This study have some limitation. One of the limitations of our study is the lack of control for certain confounding factors, such as whether the patients smoke, the use of statins, and lifestyle factors (e.g., diet and exercise). As this information was not available in our original dataset, we were unable to adjust for these relevant confounding factors. We hope that future studies can further investigate these factors to gain a more comprehensive understanding of their impact on the results.Although our cross-sectional analysis revealed a significant association between HbA1c levels and diabetic retinopathy (DR), the one-time nature of data collection limits the ability to draw causal conclusions. Additionally, the generalizability of our findings may be constrained, as the sample was drawn from a specific region and medical setting. To address these limitations, future prospective cohort studies or randomized controlled trials are needed to elucidate the underlying mechanisms linking HbA1c levels to the development of DR. Longitudinal studies could track patients’ HbA1c levels and the progression of DR over time, providing dynamic data to uncover more complex relationships. Moreover, diversifying the study sample by including patients from various regions, ethnic backgrounds, and healthcare settings will enhance the generalizability and relevance of the findings, providing a more robust scientific basis for the development of public health policies.

## Conclusion

This is the first study investigated the nonlinear association between HbA1c levels and DR. The results indicated that the risk of DR peaks at an HbA1c level of 9.4%, after which it begins to decline with further increases in HbA1c levels. These findings underscore the importance of HbA1c as a key biomarker for predicting and assessing the risk of DR. The results provide significant scientific evidence for the clinical management of diabetic patients, suggesting that healthcare providers should closely monitor HbA1c levels to reduce the incidence of DR. Furthermore, the findings offer insights for public health policy, highlighting the necessity of regular screening and intervention for diabetic patients. Future research should further explore the underlying mechanisms linking HbA1c to DR and consider individualized management strategies for different populations to effectively mitigate the burden of DR.

## Supplementary Information

Supplementary Material 1.

## Data Availability

The data generated and analyzed during this study are available in the Supplementary Information section of the manuscrip.

## Abbreviations

HbA1c: Hemoglobin A1c
DR: Diabetic Retinopathy
T2DM: Type 2 Diabetes Mellitus
BMI: Body Mass Index
SBP: Systolic Blood Pressure
DBP: Diastolic Blood Pressure
AC: Abdominal Circumference
eGFR: Estimated Glomerular Filtration Rate
ABI: Ankle-Brachial Index
OR: Odds Ratio
CI: Confidence Interval
MDRD: Modification of Diet in Renal Disease
UKPDS: UK Prospective Diabetes Study
